# Editorial Note: Comparing public willingness-to-pay for different low-carbon measures: A case study of Shenzhen, China

**DOI:** 10.1371/journal.pone.0361832

**Published:** 2026-11-02

**Authors:** The PLOS One Editors

Following the publication of this article [1], PLOS was made aware of similarities between this article [1,2, retracted in 3] and [4]. During an editorial investigation into these concerns, the *PLOS One* Editors identified potential links between the authors of [2, retracted in 3] and [4], and individual(s) involved in the peer-review of [1], raising concerns that the confidentiality of the manuscript was compromised during peer-review.

The *PLOS One* Editors did not identify any evidence that the authors of this article [1] were aware or involved in the duplicate publication of the work reported in this article. PLOS regrets that these issues were not identified during the peer-review process.
